# Inhibition of Seizure-Like Paroxysms and Toxicity Effects of *Securidaca longepedunculata* Extracts and Constituents in Zebrafish *Danio rerio*

**DOI:** 10.1021/acschemneuro.3c00642

**Published:** 2024-01-25

**Authors:** Nastaran Moussavi, Wietske van der Ent, Drissa Diallo, Rokia Sanogo, Karl E. Malterud, Camila V. Esguerra, Helle Wangensteen

**Affiliations:** † Section for Pharmaceutical Chemistry, Department of Pharmacy, University of Oslo, P.O. Box 1068, Oslo 0316, Norway; ‡ NCMM, Chemical Neuroscience Group, Centre for Molecular Medicine Norway, Faculty of Medicine, University of Oslo, Oslo 0349, Norway; § Department of Traditional Medicine, National Institute of Public Health, PB, Bamako 1746, Mali; ∥ Faculty of Pharmacy, University of Sciences, Techniques and Technologies of Bamako (USTTB), Bamako 1746, Mali; ⊥ Section for Pharmacology and Pharmaceutical Biosciences, Department of Pharmacy, University of Oslo, P.O. Box 1068, Oslo 0316, Norway

**Keywords:** *Securidaca longepedunculata*, epilepsy, toxicity, larval zebrafish, xanthones, benzoates

## Abstract

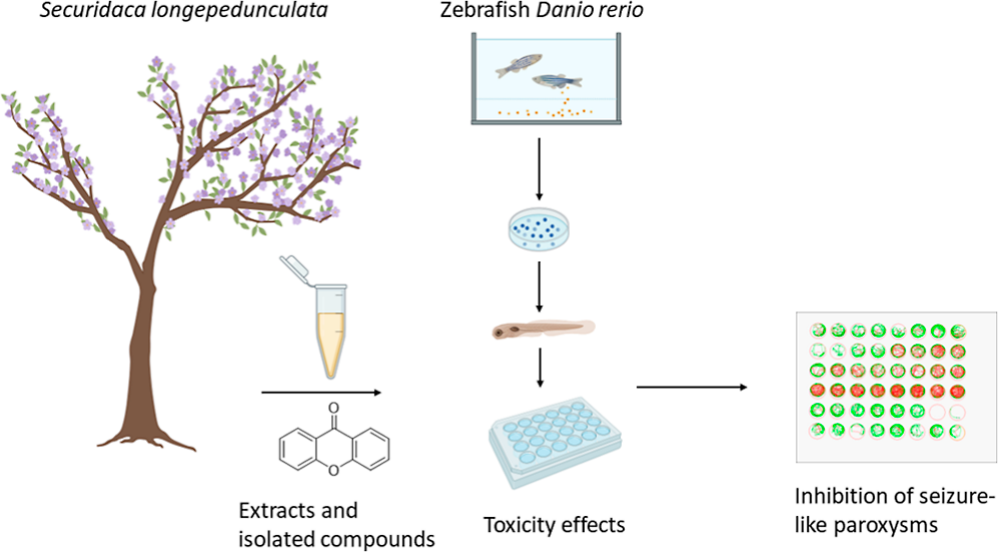

Plants used in traditional medicine in the management of epilepsy could potentially yield novel drug compounds with antiepileptic properties. The medicinal plant *Securidaca longepedunculata* is widely used in traditional medicine in the African continent, and epilepsy is among several indications. Limited knowledge is available on its toxicity and medicinal effects, such as anticonvulsant activities. This study explores the potential in vivo inhibition of seizure-like paroxysms and toxicity effects of dichloromethane (DCM) and ethanol (EtOH) extracts, as well as isolated xanthones and benzoates of *S. longepedunculata*. Ten phenolic compounds were isolated from the DCM extract. All of the substances were identified by nuclear magnetic resonance spectroscopy. Assays for toxicity and inhibition of pentylenetetrazole (PTZ)-induced seizure-like paroxysms were performed in zebrafish larvae. Among the compounds assessed in the assay for maximum tolerated concentration (MTC), benzyl-2-hydroxy-6-methoxy-benzoate (MTC 12.5 μM), 4,8-dihydroxy-1,2,3,5,6-pentamethoxyxanthone (MTC 25 μM), and 1,7-dihydroxy-4-methoxyxanthone (MTC 6.25 μM) were the most toxic. The DCM extract, 1,7-dihydroxy-4-methoxyxanthone and 2-hydroxy-1,7-dimethoxyxanthone displayed the most significant inhibition of paroxysms by altering the locomotor behavior in GABA_A_ receptor antagonist, PTZ, which induced seizures in larval zebrafish. The EtOH extract, benzyl benzoate, and benzyl-2-hydroxy-6-methoxy-benzoate unexpectedly increased locomotor activity in treated larval zebrafish and decreased locomotor activity in nontreated larval zebrafish, seemingly due to paradoxical excitation. The results reveal promising medicinal activities of this plant, contributing to our understanding of its use as an antiepileptic drug. It also shows us the presence of potentially new lead compounds for future drug development.

## 1 Introduction

Epilepsy is a neurological brain disease that involves predisposition to generate spontaneous recurring seizures, affecting more than 70 million people worldwide.^1^ Today’s pharmacotherapy does not manage to treat approximately one-third of epilepsy patients, despite the availability of numerous antiseizure medications (ASMs) in the western medical market.^2^ Furthermore, modern ASMs often have side effects.^3,4^ For this reason, the development of new and better ASMs is a subject of intensive research.^5^ The use of herbal medicines for the treatment of epilepsy is widespread throughout the world.^6^ In traditional African medicine, numerous plants are used in the treatment of epilepsy. The violet tree, *Securidaca longepedunculata* Fresen (Polygalaceae)^7^ (sometimes spelled *S. longipedunculata*), a small tree with pale gray stem bark and sweetly scented pink to purple flowers blooming in bouquets on a peduncle, is an important medicinal plant and known as the “mother of all medicines”.^8^ This tree grows mainly in seasonally dry tropical areas and has been used extensively in African traditional medicine in the treatment of a range of diseases and conditions such as malaria, rheumatism, tuberculosis, and constipation.^9,10^ Interestingly, *S. longepedunculata* has been reported to be used against epilepsy in Nigeria,^11,12^ Ethiopia,^13^ Cameroon,^14^ Zimbabwe,^15^ and Burkina Faso.^16^ A crude aqueous extract of the roots was reported to have anticonvulsant activity.^14,17^ The constituents of the plant responsible for the putative antiepileptic effects appear, however, to be unknown.

A wide range of constituents is reported in *S. longepedunculata*, including saponins, phenolic acids, benzophenones, benzoates, flavonoids, and sterols (reviewed by Mongalo et al.^9^). Of note, there is a large number of xanthones, with more than 40 reported so far. To date, no research reports are available on the effects of the xanthones found in *S. longepedunculata* on the central nervous system (CNS), although other biological effects of these compounds have been reported, e.g., cytotoxic activity against human cancer cells,^18−20^ activity against erectile dysfunction through relaxation of rabbit corpus cavernosum smooth muscle,^21,22^ and antimicrobial^23^ and antiarthritic activities.^24^ However, other xanthones have been reported as anticonvulsants and antiepileptics.^25,26^ A nonxanthone constituent, benzyl benzoate, is an acaricide and is used clinically against scabies.^27^

The knowledge of the toxicological profile of *S. longepedunculata* is limited. In ritual suicide, a decoction from the stem bark is taken orally in South Africa.^9^ It is a widespread suicide poison used by women in Zambia, Angola and the Democratic Republic of the Congo, where peeled root or root pulp is introduced into the vagina. As a hunting poison, the allegedly high toxicity of bark and roots comes into play as an arrow and fishing poison used in Nigeria, Zambia, Zimbabwe, the Democratic Republic of the Congo, Senegal, and Angola. *S. longepedunculata* is also known as murder and trial-by-order poison used in Nigeria, Cameroon, Angola and Central African Republic.^28^ To our knowledge, no investigations on the toxicity profile of *Securidaca* spp. in zebrafish have been published. Among the constituents, benzyl benzoate^29^ and benzyl 2-hydroxy-6-methoxybenzoate^30^ are reported to be toxic to brine shrimps. Additional research is necessary to balance the discrepancy between knowledge on traditional use and the potential bioactivities of compounds of *S. longepedunculata*.

The present study aims at phytochemically screening extracts through isolation and structural identification of major xanthones and related substances and exploring the toxicological activities and inhibition of antiseizure-like paroxysms of both extracts and compounds of *S. longepedunculata* bark using locomotor behavior as a readout for convulsions in a zebrafish larvae-based pentylenetetrazole (PTZ) assay for the discovery of new bioactive agents in the treatment of epileptic seizures with a good safety profile.

## 2 Results and Discussion

The present study investigates the toxicity profile and the inhibitory effects on seizure-like paroxysms induced by acute PTZ treatment of extracts and isolated compounds from the African medicinal plant *S. longepedunculata*, a plant well-known for its use against CNS disesases.^9,10^ Despite its popularity, how this plant can affect targets in the brain has not been studied. By combining phytochemistry research with pharmacological assays using the locomotor behavior as a readout for seizure-like paroxysms in a zebrafish larvae-based PTZ assay, we were able to explore the possible neurological effects of the main constituents of the lipophilic extract, the benzoates and the xanthones. Additionally, the study demonstrates zebrafish toxicity phenotypes after exposure to the test compounds. By using the zebrafish model for epilepsy and toxicity evaluation, we have obtained new knowledge about the active chemical components of *S. longepedunculata* which contribute to the understanding of its ethnopharmacological use against epilepsy and the identification of potentially new lead compounds for drug discovery.

### 2.1 Isolation of Compounds

**Figure 1:**
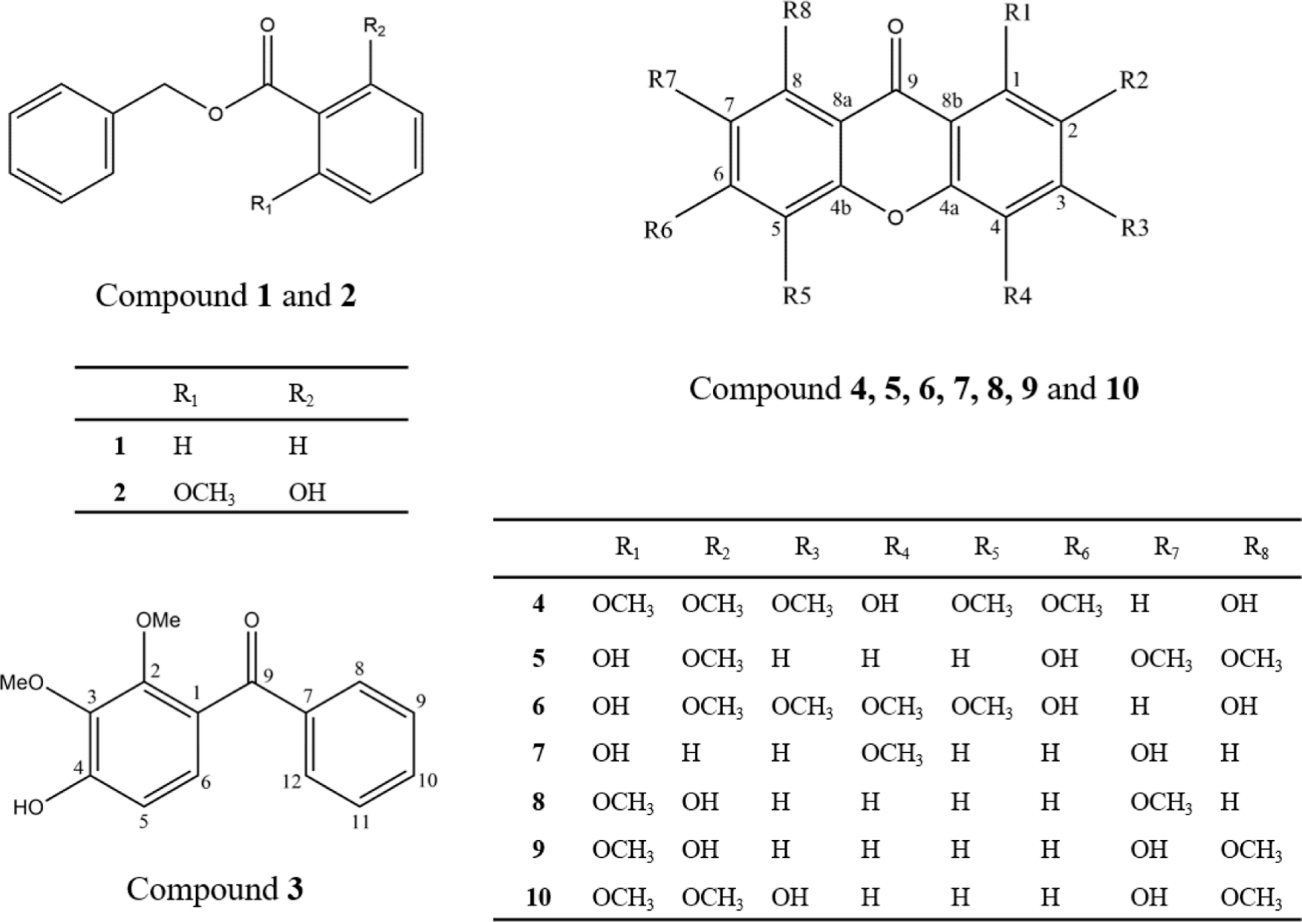
Structures of compounds **1–10** isolated from the DCM extract of *S. longepedunculata* bark.

In this work, 10 polyphenols were isolated from the bark of *S. longepedunculata* and identified by one-dimensional and two-dimensional nuclear magnetic resonance (NMR) (see Figure 1). These compounds have previously been reported in either *S. longepedunculata* or other plant species, but knowledge of their CNS effects is lacking.

Compound **1** (42.8 mg), identified as benzyl benzoate,^31^ has been reported only one time previously from *S. longepedunculata*,^32^ although it is found in many other plants. Compound **2** (33.1 mg), identified as benzyl-2-hydroxy-6-methoxy-benzoate, has previously been found in *S. longepedunculata*.^18^ Compound **3** (12 mg) was shown to consist of 2,3-dimethoxy-4-hydroxy-benzophenone by comparison with literature data.^33^ This substance has been reported previously in *S. longepedunculata*.^18^ Compound **4** (17.4 mg) was identified as 4,8-dihydroxy-1,2,3,5,6-pentamethoxyxanthone,^18^ obtained from the same plant. Compound **5** (24.9 mg), identified as 1,6-dihydroxy-2,7,8-trimethoxyxanthone, has not been previously found in *S. longepedunculata*. Its single occurrence reported so far^34^ is from *Cratoxylum cochinchinense*. Compound **6** (10.5 mg), identified as 1,6,8-trihydroxy-2,3,4,5-tetramethoxyxanthone, has previously been reported only once, namely in *S. longepedunculata*.^18^ The identity of compound **7** (16.2 mg), 1,7-dihydroxy-4-methoxyxanthone, was corroborated by comparison with literature data.^35^ This compound has been reported previously in *S. longepedunculata*;^19,36^ however, none of these provided spectroscopic data for the isolated substance. Compound **8** (7.5 mg), identified as 2-hydroxy-1,7-dimethoxyxanthone, is previously reported in *S. longepedunculata*.^22,37,38^ Our data are in good accordance with those reported.^38^ The the first time compound **9** (16 mg), identified as 2,7-dihydroxy-1,8-dimethoxyxanthone,^39^ has been reported from the genus *Securidaca*, and it is only known previously from *Cratoxylum formosanum*(39) and *Calophyllum membranaceum*.^40^ Substance **10**, 3,7-dihydroxy-1,2,8-trimethoxyxanthone, is another rare substance previously reported from the related species *Securidaca inappendiculata*(41) and *Polygala sibirica* var. *megalopha*.^42^ However, this seems to be the first time compound **10** is found in *S. longepedunculata*. NMR data of compounds **1–10** are shown in the Supporting Information.

### 2.2 Toxicological Evaluation in Larval Zebrafish

**Figure 2:**
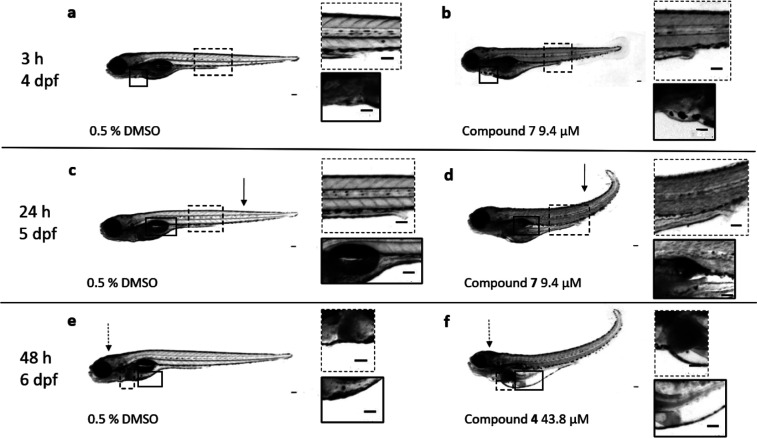
Examples of toxicological signs from morphological assessments of compounds isolated from *S. longepedunculata* on zebrafish larvae within the first 3, 24, and 48 h after treatment. (a) Larva exposed to 0.5% DMSO (control) is showing normal urogenital opening (in the dashed box) and normal heart (solid box) at 4 dpf 3 h postexposure, relative to (b) treated larva (compound **7**, 9.4 μM) displaying abnormal urogenital opening (dashed box) and abnormal edema of the heart (solid box) at 4 dpf 3 h postexposure. (c) Control larva treated with 0.5% DMSO shows normal body axis (notochord) (arrow), somites [precursors to muscle tissue (solid box)], and swim bladder (solid box) at 5 dpf 24 h postexposure, relative to figure (d) where the treated larva (compound **7**, 9.4 μM) displays abnormally curved body axis (notochord) (arrow), darkened tissues, suggestive of cell death (dashed box) and reduced swim bladder (solid box) at 5 dpf 24 h postexposure. (e) Larva as control (0.5% DMSO) shows no signs of darkened brain tissues, suggestive of cell death (dotted arrow), normal liver (dashed box), and normal yolk sac (solid box) at 6 dpf 48 h postexposure, compared to (f) treated larva (compound **4**, 43.8 μM) showing darkened brain (dotted arrow), darkened liver (dashed box) indicating cell death, and edema of the yolk sac at 6 dpf 48 h postexposure. All scale bars are presented as 100 μm.

In this in vivo assay, the maximum tolerated concentration (MTC) of the dichloromethane (DCM) and ethanol (EtOH) extracts and compounds **1**, **2**, **4**, **7** and **8** from *S. longepedunculata* was evaluated and determined by assessing their toxicological effects on wild-type (AB) zebrafish larvae (see results in Table 1). Representative and observable toxicity malformations of treated zebrafish larvae were photographically documented using a microscope camera, as highlighted images shown in Figure 2. Overview of the toxicological traits of each sample at the different time points (3, 24, and 48 h) is also displayed in Table 1.

**Table 1:** MTCs and Toxicity Phenotypes of Extracts and Compounds Isolated from *S. longepedunculata* above MTC after 3, 24, and 48 h in Zebrafish Larvae

test sample	concentration^a^	toxic phenotypes observed post treatment (percent affected)^b^			maximum-tolerated concentration (MTC)
		3 h	24 h	48 h	
EtOH extract	47.1 μg/mL	D (10%) or MN (40%), TR (80%), LP (60%), HO (40%), PE (20%)	D (100%)	D (100%)	15.7 μg/mL
	31.3 μg/mL	MN (10%), TR (50%)	MN (20%), TR (60%), HO (80%)	MN (20%), TR (60%), HO (100%)	
DCM extract	15.7 μg/mL	LP (20%), HO (20%), TR	D (40%) or LP (30%), HO (50%), TR (60%)	D (80%) or LP (50%), HO (80%), TR (60%)	11.8 μg/mL
**1**	200 μM	LP (80%), TR (60%), SB (50%)	D (100%)	D (100%)	75 μM
	100 μM	LP (50%), TR (40%), SB (10%)	D (40%) or LP (60%), TR (50%), SB (30%)	D (40%) or LP (60%), TR (60%), SB (30%)	
**2**	50 μM	LP (60%), HO (40%), PE (20%)	D (80%) or LP (60%), MN (20%), PE (20%)	D (100%)	12.5 μM
	25 μM	LP (40%), HO (30%), TR (40%)	D (40%) or LP (50%), HO (60%), TR (60%), SB (60%), SH (20%)	D (20%) or LP (50%), HO (60%), TR (70%), SB (20%), SH (10%), CS (10%)	
	18.75 μM	LP (20%), HO (10%), TR (20%)	LP (30%), HO (20%), TR (30%)	LP (30%), HO (20%), TR (30%)	
**4**	50 μM	LP (100%), HP (80%), TR (100%), SB (50%), HO (60%), PE (20%), DBT (20%)	D (100%) or LP (100%), HP (80%), TR (100%), SB (60%), DBT (20%), HA (80%), SH (60%), LN (40%), YE (30%)	D (100%)	25 μM
	43.8 μM	LP (30%), HP (20%)	LP (30%), TR (40%), HP (30%), PE (30%), SB (50%)	LP (40%), HP (50%), TR (50%), SB (50%), DBT (10%), LN (20%), YE (20%)	
	37.5 μM	LP (30%) TR (20%), HP (20%), SB (30%)	LP(30%), TR (20%), HP (20%), SB (30%), PE (20%)	LP (30%), TR (30%), HP (30%), SB (40%), PE (20%)	
**7**	12.5 μM	LP (80%), TR (50%), HO (20%), SB (60%), PE (20%), PE (40%), UO (50%)	D (100%)	D (100%)	6.25 μM
	9.4 μM	LP (50%), TR (30%), PE (20%), UO (50%), PE (20%)	LP (60%), TR (60%), PE (30%), UO (50%), CS (20%), MN (20%)	LP (60%), TR (30%), PE (30%), UO (50%), CS (20%), MN (20%), SB (60%)	
**8**	200 μM	LP (100%), TR (100%), SH (100%)	D (100%)	D (100%)	75 μM
	100 μM	TR (50%), SB (20%), SH (20%)	LP (20%), TR (50%), SH (20%), SB (10%)	LP (30%), TR (50%), SH (40%)	

a Concentrations of extracts are given in μg/mL, isolated substances in μM. At lower concentrations, toxic effects were not observed.

b Phenotypes are indicated with the following abbreviations: CS, curved spine; D, death; HA, hyperactivity; DBT, darkened brain tissue, suggestive of cell death; DLT, darkened liver tissue, indicative of cell death; HO, hypoactivity; HP, hyperpigmentation; TR, impaired motility (decreased or no touch response); LP, loss of posture; MN, muscle necrosis; PE, pericardial edema; RS, reduced swim bladder; SB, incompletely inflated or uninflated swim bladder; SH, slow heart rate; UO, abnormal urogenital opening; YE, yolk edema.

The knowledge on the toxicological profile of compounds isolated from *S. longepedunculata* is limited, while there exist in vivo toxicity studies on the aqueous, ethanolic, and methanolic root extract showing various toxic effects of all extracts.^9^ The EtOH and DCM extracts were highly toxic against the zebrafish larvae with MTC values of 15.7 and 11.8 μg/mL, respectively, compared to the pure compounds tested in this study. The EtOH extract at 31.3 μg/mL caused impaired motility and muscle necrosis of the zebrafish larvae at all time points, in addition to loss of posture after 48 h. An additional toxic abnormality, pericardial edema, occurred after 3 h of treatment when larvae were exposed to a concentration of 47 μg/mL of the EtOH extract, and after 24 h, all larvae were dead. The zebrafish larvae exposed to the DCM extract with a concentration of 15.7 μg/mL showed a consistent toxicological profile at all three time points observed as hypoactivity, impaired movement, and loss of posture, except for cases of death after 24 and 48 h. This is the first time the toxic effects of the DCM extract of *S. longepedunculata* have been assessed in a larval zebrafish assay.

Compound **1** was well tolerated by the zebrafish larvae with an MTC value of 75 μM. At higher concentrations tested (≥100 μM), the most common abnormalities observed for the treated zebrafish larvae were loss of posture, reduced touch response, and reduced swim bladder or death after 24 and/or 48 h after exposure to compound **1**. Previously, one toxicity study on zebrafish larvae (AB strain) has been performed on compound **1** showing toxic effects at higher concentrations, in accordance with our results.^43^ Today, benzyl benzoate (compound **1**) is a well-known agent used as a sweetening additive and carrier solvent in foods, stabilizer in perfumes and pharmaceuticals due to its low volatility, insecticide combating pests in agricultural production, and in the treatment of scabies since 1937.^44−46^ The MTC value of compound **2** was determined to be 12.5 μM. Compound **2** was not well tolerated by treated larvae, displaying a trend of loss of posture, reduced touch response, and hypoactivity, across all three time points above a concentration of ≥18.75 μM. Pericardial edema, reduced swim bladder, and slow heart rate of the zebrafish larvae were observed at concentrations above 25 μM, whereas muscle necrosis was developing after 24 h of treatment with 50 μM of compound **2**. Compound **2** has been reported to have cytotoxic effects in vitro.^30,47^ In an in vivo assay, compound **2** has also been reported as toxic to brine shrimp larvae.^30,47^ One in vitro study on benzyl 2-hydroxy-6-methoxybenzoate (compound **2**) and methyl 2-hydroxy-6-methoxybenzoate has revealed inactivity against various bacterial and fungal species.^19^ Zebrafish larvae treated with compound **4** revealed a diverse array of abnormal phenotypes with an MTC value of 25 μM. Treatment of the larvae with a concentration of 37.5 μM resulted in loss of posture, hyperpigmentation, reduced touch response, and reduced swim bladder when examined after 24 and 48 h. At higher concentrations (50 μM), the zebrafish larvae were strongly affected by compound **4**, being subject to additional pericardial edema and slow heart rate, showing darkened liver and brain tissues, suggestive of cell death, until 100% death occurred after 48 h, see Figure 2. Interestingly, larvae treated with compound **4** (50 μM) were first hypoactive within the first 3 h and then hyperactive when examined after 24 h, before occurrence of death after 48 h. Compound **4** has been reported only once as a natural product. It was without cytotoxicity on human pancreatic cancer cells.^18^ Compound **7** was found to be the most toxic compound tested in this study, with an MTC value of 6.25 μM. Curved spine, abnormal urogenital opening, pericardial edema, muscle necrosis, and reduced swim bladder were found as distinct aberrant phenotypes in larvae exposed to 9.4 μM of compound **7**, see Figure 2. Compound **7** has been reported to have cytotoxic effects in vitro.^20,36,48^ As one of the least toxic compounds in this study, compound **8** demonstrated few visible phenotypical changes in larvae exposed to concentrations above the MTC-value (75 μM). Slow heart rate and reduced touch response appeared to be the most common toxic effects observed in larvae when exposed to higher concentrations, 100 and 200 μM. Compound **8** has previously shown moderate cytotoxic activity and no antiviral activity toward influenza virus.^49^ In another study, compound **8** did not exhibit potent cytotoxic activity against the growth of different human tumor cell lines.^20^

Studies on benzoates and xanthones isolated from *S. longepedunculata* are sparse. 2,3-Dimethoxy-4-hydroxy-benzophenone (compound **3**) has showed low cytotoxicity against pancreatic cancer PANC-1 cells,^18^ and in another study, **3** exhibited antiplasmodial activity.^50^ To date, no in vivo studies have been performed on 1,6-dihydroxy-2,7,8-trimethoxyxanthone (compound **5**), 2,7-dihydroxy-1,8-dimethoxyxanthone (compound **9**), and 3,7-dihydroxy-1,2,8-trimethoxyxanthone (compound **10**). 1,6,8-Trihydroxy-2,3,4,5-pentamethoxyxanthone (compound **6**) has displayed cytotoxic activity against human pancreatic cancer PANC-1 cells under nutrient-deprived conditions.^18^ Furthermore, other xanthones previously isolated from *S. longepedunculata*, 1,6-dihydroxy-2,3,4,5,8-pentamethoxyxanthone,^18^ and muchimangin B have induced apoptotic-like cell death of human pancreatic cancer PANC-1 cell line.^19,51^ A xanthone extract from *Garcinia mangostana* has shown toxicity to zebrafish embryos at concentrations of 62.5 μg/mL and higher.^52^ The substances in our experiments were, however, not tested. A xanthone from *G. mangostana*, α-mangostin, has been found to be toxic and teratogenic in zebrafish embryos.^52−57^

It was not possible to determine the structure–activity relationship (SAR) of either the benzoates or xanthones tested in this toxicological assay. More data are needed to enable the determination of the chemical group accountable for the toxic effects in larval zebrafish.

To our knowledge, this is the first report on the MTCs of compounds **1**, **2**, **4**, **7,** and **8** and extracts isolated from *S. longepedunculata* on zebrafish larvae.

### 2.3 Inhibition of PTZ-Induced Seizure-Like Paroxysms in Larval Zebrafish

Anticonvulsant, anxiolytic, and sedative effects of an aqueous root extract of *S. longepedunculata* on mice have been reported.^9^ One in vitro study has revealed the ability of xanthones, such as garcinone, γ-mangosteen, and gartanin, isolated from *G. mangostana*, to penetrate the blood brain barrier (BBB). In addition, these three xanthones are suggested as anti-Alzheimer agents since they cause antiamyloid plaque formation through inhibitory effects against Aβ42 in *Escherichia coli* cells, toward Aβ42 self-induced aggregation in a tube and against BACE1 (β-site amyloid precursor protein-cleaving enzyme 1).^58^ One newly patented compound, 6-hydroxy-1,2,3,7-tetramethoxyxanthone, has been reported to improve depression by increasing the number of hippocampal neural stem cells.^59^ 1,7-Dimethoxy-2-hydroxy-xanthone and 1,3,6,8-tetrahydroxy-2,5-dimethoxyxanthone have exhibited activity against erectile dysfunction through relaxation of rabbit corpus carvenosal smooth muscle,^21,22^ and some other xanthones have showed moderate antimicrobial activity.^23^

**Figure 3:**
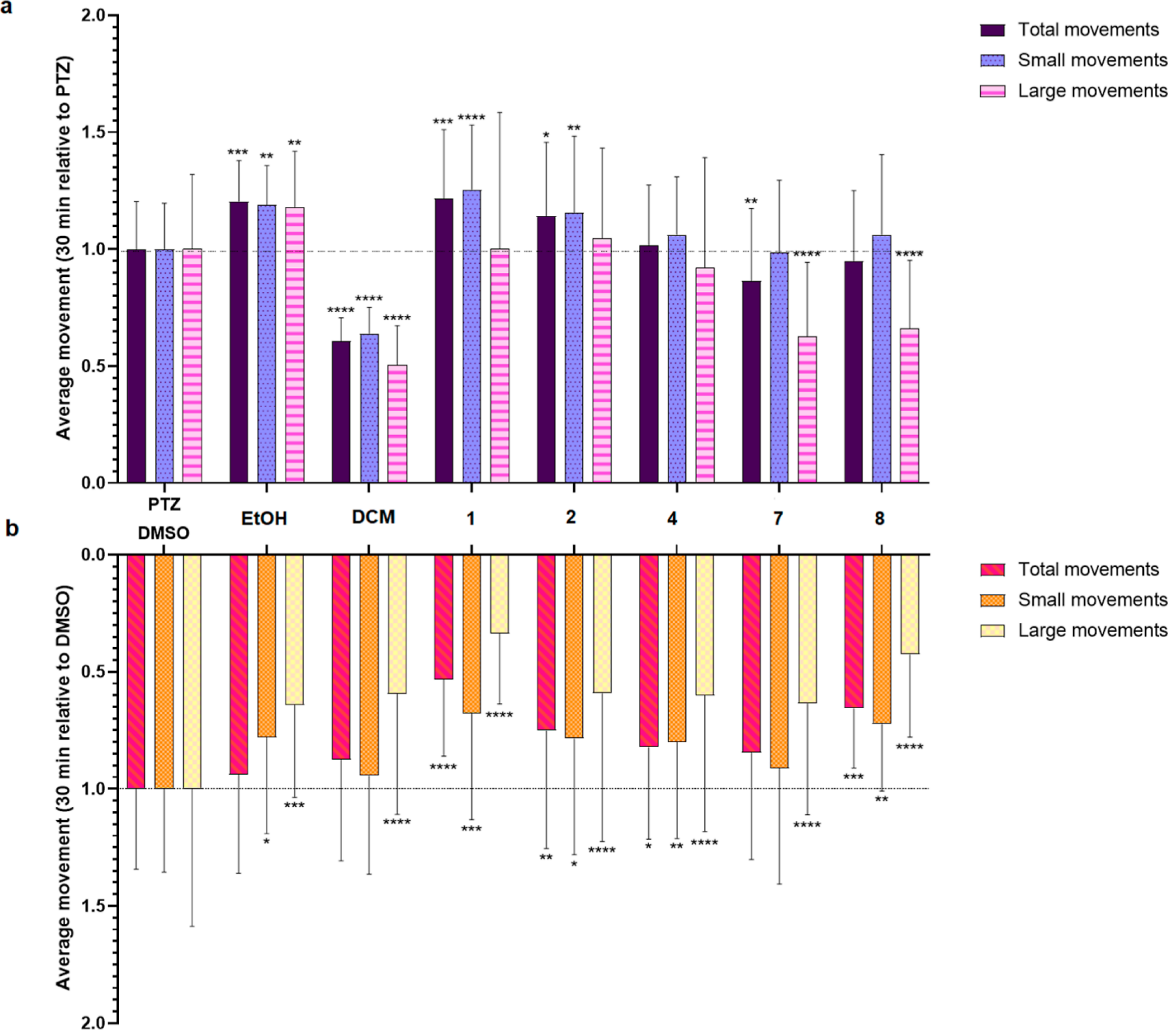
Zebrafish behavioral assay showing inhibition of seizure-like paroxysms (a) and hypoactivity (b) of *S. longepedunculata* extracts and compounds after 30 min tracking period. The concentration of *S. longepedunculata* compounds and extracts used in both graphs (a,b) was equal to the MTC found after their toxicological evaluation in larval zebrafish (see Section 2.2). The final concentration of DMSO was 0.5% per well. (a) Average distance traveled (mm/30 min) by zebrafish larvae (*y*-axis) treated with PTZ + 0.5% DMSO (vehicle). Data normalized against the PTZ-control (final concentration 20 mM). (b) Average distance traveled (mm/30 min) by zebrafish larvae treated with 0.5% DMSO (*y*-axis). Data normalized against the DMSO control. Data were analyzed using two-way ANOVA with multiple comparisons. Results are expressed as means ± standard deviation (SD) of three to six separate experiments. Sample (*n* = 12), sample + PTZ (*n* = 12), PTZ (*n* = 8), fish water control (*n* = 8), and fish water + 0.5% DMSO (*n* = 8). *n*—represents the total number of larvae. **** = *p* < 0.0001, *** = *p* < 0.001, and ** = *p* < 0.01. * = <0.05, compared to controls; PTZ- or DMSO-treated larvae. Fish water and 0.5% DMSO were used as negative control (untreated larvae).

Extracts and compounds **1**, **2**, **4**, **7,** and **8** isolated from *S. longepedunculata* were tested for their ability to reduce PTZ-induced convulsive-like movements in zebrafish larvae at 5 dpf. The MTC of compounds and extracts of *S. longepedunculata* was determined before the evaluation of anticonvulsant activity through the inhibition of seizure-like paroxysms induced by acute PTZ treatment in larval zebrafish. This behavioral analysis was based on the tracking of PTZ-induced convulsion-like activities of chronically pretreated and nontreated zebrafish larvae detected by video recordings.^60,61^ The larval locomotor behavior was tracked and divided into four categories: inactivity (<4 mm/s), small movements (4–20 mm/s), large movements (>20 mm/s), and total movements (see Supporting Information with examples of the different movements). The rationale for measuring the basic motor patterns, large and small movements, was to make quantitative descriptions of larval behavior contributing to a better understanding of possible antiseizure effects of *S. longepedunculata*. Large movements have been associated with tonic-clonic like seizures observed as bursts of swimming. Small movements have been described as myoclonic-like behavior characterized as increased orofacial movements and fin fluttering.^62^ The total movements was defined as equal to the average of the sum of both small and large movements expressed in millimeters per second. As shown in Figure 3, all types of movements are shown in mm within a period of 30 min as averages (summary of 5 min time bins).

As shown in Figure 3a, the DCM extract was the most active in terms of significantly reducing all types of PTZ-induced locomotor activity, both large and small movements, in zebrafish larvae after 20–22 h of incubation at determined MTC (11.8 μg/mL). The major reduction of all types of movements with regard to the DCM extract might reveal synergistic effects and/or the existence of other potent compounds in the extract not tested in this assay. Pretreatment with compounds **7** and **8** isolated from the DCM extract exhibited strong reduction (30–35%) in PTZ-induced large movements of larval zebrafish, tested at their respective MTC-values at 6.25 and 75 μM. Regarding the molecular structure of the compounds, fewer hydroxyl or methoxyl substituents on the xanthone backbone might appear to be favorable for anticonvulsant activity in treated larvae. Substance **4** seemed to be less active in this respect, although a significant difference was not observed. Substances **7** and **8** have a lower degree of substitution than compound **4**.

As shown in Figure 3b, displaying treatment without PTZ, both extracts and compounds of *S. longepedunculata* were able to reduce both large and small movements of larval zebrafish after 20–22 h of incubation relative to the DMSO control, showing the activity of *S. longepedunculata* itself. Treatment with compound **1** resulted in hypoactivity, with the highest drop of both large (65% reduction) and small (45% reduction) movements. In contrast, the results for compound **1** corresponded inversely with PTZ-treated zebrafish larvae regarding small movements, indicating the inability to reduce small movements of PTZ-induced larval locomotor behavior, see Figure 3a.

The EtOH extract contributed significantly to increased larval activity of both large and small locomotor activities in the PTZ-treated larvae compared to the PTZ-control (Figure 3a). The increased movements in PTZ-treated larval zebrafish related to compounds **1** and **2** (small movements) and the EtOH extract (large and small movements) might be triggering the γ-amino butyric acid (GABA) paradox, leading to hyperexcitation. To determine this in the future, additional tests such as acoustic startle response with extra control would need to be performed. One comprehensive study has shown that some CNS depressants, such as benzodiazepines, which are related to sedation, also cause paradoxical excitation in zebrafish.^63^ Paradoxical excitation is defined as decreased neuronal activity but with paradoxically increased activity in the caudal hindbrain, causing escalated motor activity. Substances that cause paradoxical excitation are agonists or positive allosteric modulators (PAMs) of GABA receptors. It must be noted, however, that the behavioral assays performed in the study by McCarroll^63^ used the acoustic startle response as the primary readout and is therefore different from seizure-like paroxysms elicited by PTZ. Thus, additional follow up studies comparing the larval response to PAMS or GABA agonists with the responses to compounds **1**, **2** and the EtOH extracts are required in order to confirm activation of the GABA paradox. Increased neuronal activity in the brain can be shown with GCaMP imaging and whole brain imaging of pERK, which are potentially useful studies for broadening the understanding of seizuregenic phenotypes beyond simple locomotor assays.^64,65^ There exists ASMs such as carbamazepine, levetiracetam, and zonisamide with no proven efficacy in pharmacological antiseizure response observed in PTZ-treated zebrafish, meaning that compounds **1** and **2** and the EtOH extract are still interesting as anticonvulsants as they may act via mechanism(s) other than decreasing PTZ-induced seizures in zebrafish. However, the drugs, zonisamide and levetiracetam, are reported with proven efficacy in PTZ-induced seizures in rodents. Also, valproate, diazepam, tiagabine, and ethosuximibe are effective as ASMs in both PTZ-treated zebrafish and rodents.^66^ The ideal result of a compound acting as an antiepileptic drug in this assay would possibly show a reduction of movements in PTZ-treated fish and no reduction in non-PTZ-treated fish, such as for the DCM extract (small movements). Some reduction in non-PTZ treated fish is also of interest, as this could indicate sedative activity or locomotor impairment. If there is a wide active concentration range for a compound, then ideally, a concentration where there is still an anticonvulsant activity of the compound and no or minimum “sedative” activity is ideal.

PTZ-induced locomotor assays, which include larval zebrafish, are widely used in both pharmacological and genetic screens primarily for the discovery of novel natural compounds and small molecules acting as antiseizure agents.^66−68^ After 20–22 h of incubation of the samples of *S. longepedunculata*, 5 days postfertilization (dpf) zebrafish larvae were exposed to an acute dose of PTZ (20 mM). Shortly after the addition of PTZ in the fish water, behavioral changes ascribed to acute seizures are observed in adult zebrafish.^66^ Behavioral responses of zebrafish larvae treated acutely with PTZ are elicited and displayed as spasms and hyperactivity along the periphery of the well (stage I), followed by rapid circular swimming often called “whirlpool-like” swimming or corkscrew swimming due to their helical paths of swimming (stage II). At higher PTZ concentrations, seizure-like behavior is observed as sudden jerky movements and brief pauses switching between them, as well as periods of loss of posture and body-stiffening named freezing (stage III).^66,69,70^ As a proconvulsant drug, PTZ acts as a GABA_A_ receptor blocker.^69^ Although the mechanism of action of PTZ is not fully defined, one previous study on zebrafish larvae has shown that PTZ causes epileptic seizures resembling clonus-type convulsions in mammals as well as a dose-dependent series of stereotypical behaviors.^70^ Since the distance traveled by larval zebrafish does not cover all seizure behaviors, secondary bioassays such as brain activity recordings (local field potential recordings in larval zebrafish brains) and studies on mutant zebrafish lines would be highly relevant in future studies. Isolation of further components from both extracts in enough amounts would be relevant for the investigation of new bioactive agents in the treatment of epileptic seizures. Additional behavioral assays, such as thigmotaxis and acoustic startle response, would also be of interest to be performed in future studies.

## 3 Conclusions

In this study, two benzoates (**1**, **2**), one benzophenone (**3**) and seven xanthones (**4–10**), were isolated from the DCM extract from *S. longepedunculata*, an African medicinal plant used against epilepsy. Compounds **2**, **4,** and **7** displayed the most toxic properties in zebrafish larvae, with MTC values of 12.5, 25, and 9.4 μM, respectively. The highest degree of inhibition of seizure-like paroxysms induced by acute PTZ was found in the DCM extract (small and large movements) and compounds **7** and **8** (large movements). The more highly substituted compound **4** was less active with regard to the reduction of large movements in the zebrafish locomotor activity assay. These findings suggest that the DCM extract as well as compounds **7** and **8** exert promising anticonvulsant-like activity and that they may have a potential to be utilized as a medicinal plant resource for the treatment of epilepsy. The dual effect in the reduction of larval locomotor activity without PTZ treatment and increased locomotor activity in PTZ treated larvae sheds light on the bioactivity of the EtOH extract (small and large movements) and compounds **1** and **2** (small movements) to affect sedation and cause paradoxical excitation in vivo in zebrafish, characterizing them as interesting candidates of GABA_A_ receptor ligands and modulators.

## 4 Materials and Methods

### 4.1 Plant Material

The bark of *S. longepedunculata* was collected in Bamako, Mali (coordinates 12°38′21″N 8°0′10″W) in 2017. The plant material was identified by botanists at the Department of Traditional Medicine (DMT), Bamako, Mali. A herbarium specimen (no. 2220) is deposited at the DMT. A voucher sample of the bark is kept (no. 122) in the Department of Pharmacy, University of Oslo, Norway. The bark was cut into small pieces and air-dried.

### 4.2. General Phytochemistry Methods

4.2

One- and two-dimensional NMR spectra were recorded in CDCl_3_ on a Bruker AVIII400 or a Bruker AVII600 instrument (Bruker, Rheinstetten, Germany). Tetramethylsilane (TMS) (Sigma-Aldrich, St. Louis, MO, USA) was used as a reference. Flash chromatography was performed on a Biotage Select Flash instrument equipped with Biotage Sfär silica (Si) gel columns (Biotage, Uppsala, Sweden) or on a VersaFlash system (Supelco, Bellefonte, PA, USA) with Versapak normal phase Si gel columns. Open column chromatography was performed on Sephadex LH20 (Pharmacia, Uppsala, Sweden) or MCI gel CHP20P (Supelco). Fractions were combined as indicated by their UV absorbance or by analytical TLC.

Analytical TLC was carried out on normal phase silica gel 60F_254_, 0.2 mm thick layers (Merck, Darmstadt, Germany). Spots were visualized by irradiation with short-wave (254 nm) and long-wave (366 nm) UV rays (UVGL-58 instrument, Ultra-Violet Products, Upland, CA, USA) and spraying the TLC-plates with a 1% solution of Ce(SO_4_)_2_ in 10% aqueous H_2_SO_4_, followed by heating (105 °C, 5 min). Preparative TLC was done on Si gel 60F_254_ plates, 0.5 mm thickness (Merck), with visualization by UV irradiation. Some fractions were purified by centrifugally accelerated TLC (CA-TLC) on a Chromatotron model 7924T (Harrison Research, Palo Alto, CA, USA) on gypsum-containing Si gel (Merck).^71^ All chemicals and solvents were of the highest quality grade.

### 4.3 Extraction of Plant Material

The dried bark was pulverized using a blender (RAW Pro X1500) to a fine powder (1 mm). The powdered material (595 g) was mixed with diatomaceous earth (Thermo Scientific, Waltham, MA, USA) (4:1 v/v), loaded in 100 mL steel cartridges, and extracted on an accelerated solvent extraction system (ASE 350; Dionex, Sunnyvale, CA, USA) with dichloromethane (DCM) followed by 80% ethanol (EtOH). The extraction process consisted of a preheating period of 5–7 min and 5 min of static extraction per cycle at 60 °C under a pressure of 1600 PSI. The extraction was performed three times. Solvents (DCM or 80% EtOH) were evaporated on a rotavapor, followed by an oil pump (Edwards, Crawley, UK). A dark brown sticky mass (11 g, 1.79%) was obtained from the DCM extract, and a light brown friable mass (74 g, 12.4%) was obtained from the 80% EtOH extract.

### 4.4 Isolation of Low Molecular Weight Compounds

The DCM extract (9.3 g, 86.6% of the total amount) was redissolved in DCM (2 × 30 mL), filtered, applied to a Biotage Sfär Duo silica gel column (100 g), and fractioned with a gradient of DCM–ethyl acetate (EtoAc)–methanol (MeOH) (0–100%), detected by UV absorbance at 220 and 254 nm to give 13 major fractions (SLDCM1–13). All fractions were subjected to 1H NMR spectroscopy. Fraction 1 (SLDCM1, 0.65 g) was rechromatographed on a Biotage Sfär HC silica gel column (50 g) and fractionated with a gradient of EtOAc–*n*-heptane (5–100%), detected by UV absorbance at 254 and 280 nm to give 22 fractions. As indicated by analytical TLC (mobile phase 95:5, *n*-heptane/EtOAc), the collected fractions were combined into 10 subfractions (SLDCM1-F-1 to F-10), fraction SLDCM1-F2 giving benzyl benzoate (**1**) and fraction SLDCM1-F4 giving benzyl 2-hydroxy-6-methoxy-benzoate (**2**).

Fraction 4 from the first Biotage column (SLDCM4, 1.6 g) was chromatographed over Sephadex LH-20 (4.5 × 28 cm) with MeOH as eluent, giving nine fractions (SLDCM4-SEP1 to SEP9). SLDCM4-SEP3 (350 mg) was further separated on silica gel (Versaflash column, 40 × 75 mm) with a DCM–EtOAc gradient. Fractions were combined, as indicated by TLC. SLDCM4-SEP3 V2 from this step was purified by chromatography over MCI CHP20P gel (2.5 × 25 cm) with MeOH as eluent, yielding 4-hydroxy-2,3-dimethoxybenzophenone (**3**). SLDCM4-SEP3 V3 was purified by CA-TLC with a DCM-EtOAc gradient as the mobile phase, furnishing 4,8-dihydroxy-1,2,3,5,6-pentamethoxyxanthone (**4**). SLDCM4-SEP3 V4 was purified similarly, giving 1,6-dihydroxy-2,7,8-trimethoxyxanthone (**5**). SLDCM4-SEP5 was chromatographed (Versaflash) as described above. SLDCM4-SEP5 V2 from this separation was further purified by preparative TLC with DCM-EtOAc 95:5 as the mobile phase, giving 1,6,8-trihydroxy-2,3,4,5-tetramethoxyxanthone (**6**). SLDCM4-SEP8 was similarly chromatographed, and SLDCM4-SEP8 V5 was purified by preparative TLC with DCM–EtOAc (9:1) as the mobile phase, yielding 1,7-dihydroxy-4-methoxyxanthone (**7**).

Fraction 5 (SLDCM5, 1.1 g) was purified on an MCI CHP20P column (2.5 × 25 cm) with a stepwise gradient from 50% aqueous MeOH to pure MeOH, followed by increasing amounts of EtOAc, ending with 50% EtOAc in MeOH. SLDCM5-MCI7 from this column was purified by CA-TLC (gradient: DCM to EtOAc). SLDCM5-MCI7CA1 from CA-TLC was subjected to preparative TLC (DCM–EtOAc, 17:3), giving 2-hydroxy-1,7-dimethoxyxanthone (**8**).

Fraction 7 (SLDCM7, 180 mg) was subjected to CA-TLC (gradient: DCM to EtOAc as the mobile phase) giving eight fractions. SLDCM7-CA4 was purified by preparative TLC with DCM–EtOAc (4:1) as the mobile phase, giving 2,7-dihydroxy-1,8-dimethoxyxanthone (**9**). SLDCM7-CA5–7 were combined and rechromatographed similarly by CA-TLC. SLDCM7-CA5-7CA3-4 from this was purified by preparative TLC (mobile phase DCM–EtOAc, 3:1), giving 3,7-dihydroxy-1,2,8-trimethoxyxanthone (**10**).

All substances were identified by NMR spectroscopy (^1^H NMR, 13C NMR, and 2D spectra). Compounds **1**, **2**, **4**, **7**, and **8** were obtained in sufficient amounts and purity for biological assays.

### 4.5 Animals

Wildtype adult zebrafish (genetic strain AB, Centre for Molecular Medicine Norway (NCMM), Oslo Science Park, Norway) were housed and maintained under controlled environmental conditions at temperatures of 28 °C under 14 h light and 10 h dark cycle (standard aquaculture conditions) as previously described.^72,73^ Three times a day, adult zebrafish were fed, twice daily with commercial dry feed (Gemma Micro 300, Skretting, Norway) and once daily with live brine shrimp (*Artemia*). After natural spawning, zebrafish eggs were collected from static tanks and transferred to Petri dishes for further handling and development for the purpose of the experiments. Only fertilized embryos were selected and reared in an incubator at 28 °C. Both embryos and larval zebrafish were raised in artificial fish water (E3 medium) containing 1.5 mmol/L HEPES, 17.4 mmol/L NaCl, 0.21 mmol/L KCl, 0.12 mmol/L MgSO_4_, and 0.18 mmol/L Ca[NO_3_]_2_ at pH 7.6 in the incubator.^74^ Zebrafish larvae were washed off with embryo medium from the first day of selection of eggs until the experiment day (4 or 5 dpf).

### 4.6 Toxicological Evaluation in Larval Zebrafish

The MTCs of *S. longepedunculata* extracts and compounds **1**, **2**, **4**, **7**, and **8** (dissolved in DMSO, 0.5% final concentration) were investigated by using zebrafish larvae of 4, 5, and 6 days postfertilization (dpf). The addition of test substance to the larval zebrafish started at 4 dpf, as this is the latest possible time point before the larvae are considered as animals and subject to regulations for animal experimentation. Moreover, the BBB in zebrafish is open from 3 dpf to 10 dpf with no permeability from 11 dpf;^75^ thus, the permeability of *S. longepedunculata* extracts and compounds across the BBB is possible at 4 dpf. At this early stage of the BBB’s development, there exist some degree of exclusion of substances from this barrier; these are however mostly identified as larger compounds and peptides.^76^ Within the larval stage of 4 dpf, major brain subdivisions have been formed, and differentiated neural subtypes (such as Schwann cells, astrocytes, and oligodendrocytes) have developed.^77^ The zebrafish larvae were incubated for 3, 24, and 48 h at 28.5 °C at constant light prior to toxicological evaluation. When raised under a static dark–light cycle (14 h light and 10 h dark cycle), the larvae showed the most consistent basal activity levels as previously described.^78^ Larval fish were arranged in a 24-well plate. Five zebrafish larvae and 5 μL of the test sample were added in 995 μL of the embryo medium to each well. The MTC was estimated to be the highest concentration of a sample in the embryo medium that showed no signs of phenotypical or locomotor abnormalities nor death after 3, 24, or 48 h incubation periods. Each of the larvae was examined for signs of toxicity as well as acute (3 h) and chronic (24 and 48 h) locomotor impairment under a microscope (Leica MDG41, Singapore). The following phenotypes of larvae were scored by binary notations as toxicological characterizations: body deformities (i.e., changes in pigmentation, jaw defects, and body axis), exophthalmos (bulging eyes), hypoactivity (i.e., decreased or no touch response), organ dysfunction (slow heartbeat, deflated swim bladder, abnormal blood flow, pericardial edema, and yolk sac edema), hemorrhaging (i.e., pericardial edema), loss of posture, darkened liver and brain tissues (suggestive of cell death), and death.^79^ Darkening of mentioned tissues, which are normally clear, were scored with reference to the study of He et al. 2013, showing the darker brown or gray coloration and texture of liver tissues becoming amorphous, suggesting degeneration and/or necrosis.^80^ Normal visible phenotypes of zebrafish larvae are shown in Figure 2. In order to evaluate the effects of the samples on the larval muscle function and performance, their escape responsiveness was determined.^81^ Escape behavior registered as “no visible response” was defined as an absent visible covered distance or movement upon light touch stimulus of the zebrafish tail tip with a plastic needle. Spontaneous movements constituting twice the body length of the larva were considered as normal behavior. Shorter movement distances or delayed responses observed were defined as decreased or impaired touch response. The assay was conducted in a minimum of three independent experiments with duplicates for each sample per experiment. A series of various concentrations (1:1 dilutions) per sample were tested on the larvae. At least 30 larvae were used in total per sample to determine the MTC value. Two control groups, embryo medium (negative control) and 0.5% DMSO (vehicle control), and triplicates of these were applied per 24-well plate. Data from the experiments were not recorded if 1 or more larva(s) of the controls were abnormal or dead.

### 4.7 Inhibition of Seizure-Like Paroxysms in a Locomotor Tracking System in Larval Zebrafish

Inhibition of seizure-like paroxysms of *S. longepedunculata* extracts and compounds was assessed by measuring the level of reduction of larval movements induced by PTZ, as previously described,^79^ with minor modifications. Prior to the behavioral analysis, 4 dpf larvae were treated with samples in a total volume of 1000 μL of fish water preincubated for 20–22 h in the dark. Five larvae were added to each well of a 24-well plate in three replicates, meaning that 15 larvae were utilized per concentration of a sample tested. The respective MTC values for each sample used in this assay originated from the toxicological evaluation assay, as previously described. To a 48-well plate was transferred one 5 dpf treated larva to each well filled with 200 μL of fish water. Twelve larvae were used per test sample (extract or compound) per experiment, and eight larvae were used for each control (fish water and 0.5% DMSO). The plate with treated larvae was placed in an experimentally sound-attenuated room. The larvae were allowed to habituate for 15 min in order to acclimate to the environmental conditions of the recording chamber and the experiment room. The chemoconvulsant, PTZ, was added to each well (sample wells and PTZ-control wells) obtaining a final concentration of 20 mM.^79,82,83^ By bathing the larval zebrafish in a solution of PTZ and fish water, seizures are induced after absorption of the chemoconvulsant through the skin, gills, and gastrointestinal tract from the surrounding medium, eventually reaching and affecting the brain.^79^ After 5 min of habituation, an automated video tracking system registered the larval activity utilizing Zebrabox hardware and Zebralab software (Viewpoint, Lyon, France) to analyze their behavior for 30 min with 5 min time bins.^79,83^ The distance traveled by the larva was recorded and quantified in millimeters (mm). All experiments were conducted in the time period between 10:00–17:00. The Zebrabox is a noise-canceling chamber, providing a high-resolution infrared digital video camera that allows automated observation and tracking of larval movements. The Zebralab software is used to analyze the video-tracked larval locomotor activity.^60^ Tracking data from each experiment was normalized against the control values (20 mM PTZ or 0.5% DMSO) and set at 1. Results from the tracking assays were assembled from three to six replicates of independent experiments.

### 4.8 Statistical Analysis

After exporting and processing the data from the locomotor movement tracking assay into an Excel format, statistical analyses were performed with GraphPad Prism 9 software. Data from the MTC assay were registered and processed in Excel. Data from each experiment of the locomotor movement tracking assay were normalized against the values from control samples; vehicle (DMSO) + PTZ controls or DMSO controls. The control samples were used as references for each sample tested. Subsequently, the normalized data from all replicate runs were pooled together. Analysis of the electrographic data and the average total movement within 30 min were performed using two-way ANOVA. For comparison of multiple samples, Dunnett’s test was applied.

## Data Availability

Data will be made available upon request.

## Supporting Information Available

NMR data for isolated compounds and figure illustrating the different movements in the locomotor activity tracking assay (PDF)

## Notes

All experimental and husbandry procedures of the zebrafish were carried out in accordance with the ARRIVE guidelines and the National Institute of Health Guidelines for the Care and Use of Laboratory Animals and the European Community Council Directive of November 2010 for Care and Use of Laboratory Animals (Directive 2010/63/EU). All of the experiments were performed under the approval of The Norwegian Food Safety Authority (FOTS-ID 23935).

## Abbreviations

ASM: antiseizure medication
BBB: blood brain barrier
CA-TLC: centrifugally accelerated thin-layer chromatography
CNS: central nervous system;
DCM: dichloromethane
dpf: days postfertilization
EtOAc: ethyl acetate
EtOH: ethanol
GABA: gamma amino butyric acid
MeOH: methanol
MTC: maximum tolerated concentration
NMR: nuclear magnetic resonance
PTZ: pentylenetetrazole

## References

[ref1] Fisher R. S.; Acevedo C.; Arzimanoglou A.; Bogacz A.; Cross J. H.; Elger C. E.; Engel J. Jr.; Forsgren L.; French J. A.; Glynn M.; Hesdorffer D. C.; Lee B. I.; Mathern G. W.; Moshé S. L.; Perucca E.; Scheffer I. E.; Tomson T.; Watanabe M.; Wiebe S. ILAE official report: A practical clinical definition of epilepsy. Epilepsia 2014, 55 (4), 475–482. 10.1111/epi.12550. 24730690

[ref2] Tang F.; Hartz A. M. S.; Bauer B. Drug-Resistant Epilepsy: Multiple Hypotheses, Few Answers. Front. Neurol. 2017, 8, 301 10.3389/fneur.2017.00301. 28729850 PMC5498483

[ref3] Mutanana N.; Tsvere M.; Chiweshe M. K. General side effects and challenges associated with anti-epilepsy medication: A review of related literature. Afr. J. Prim. Health Care Fam. Med. 2020, 12 (1), e1–e5. 10.4102/phcfm.v12i1.2162. PMC7343956 32634006

[ref4] Akyüz E.; Köklü B.; Ozenen C.; Arulsamy A.; Shaikh M. F. Elucidating the Potential Side Effects of Current Anti-Seizure Drugs for Epilepsy. Curr. Neuropharmacol. 2021, 19 (11), 1865–1883. 10.2174/1570159X19666210826125341. 34525933 PMC9185788

[ref5] Khan A. U.; Akram M.; Daniyal M.; Akhter N.; Riaz M.; Akhtar N.; Shariati M. A.; Anjum F.; Khan S. G.; Parveen A.; Ahmad S. Awareness and current knowledge of epilepsy. Metab. Brain Dis. 2020, 35 (1), 45–63. 10.1007/s11011-019-00494-1. 31605258

[ref6] Auditeau E.; Chassagne F.; Bourdy G.; Bounlu M.; Jost J.; Luna J.; Ratsimbazafy V.; Preux P. M.; Boumediene F. Herbal medicine for epilepsy seizures in Asia, Africa and Latin America: A systematic review. J. Ethnopharmacol. 2019, 234, 119–153. 10.1016/j.jep.2018.12.049. 30610931

[ref7] World Flora Online. *Securidaca longepedunculata* Fresen. https://wfoplantlist.org/plant-list/taxon/wfo-0000503535-2022-12?page=1 (accessed Dec 20, 2023).

[ref8] Adebiyi R. A.; Elsa A. T.; Agaie B. M.; Etuk E. U. Antinociceptive and antidepressant like effects of *Securidaca longepedunculata* root extract in mice. J. Ethnopharmacol. 2006, 107 (2), 234–239. 10.1016/j.jep.2006.03.017. 16647235

[ref9] Mongalo N. I.; McGaw L. J.; Finnie J. F.; Staden J. V. *Securidaca longipedunculata* Fresen (Polygalaceae): A review of its ethnomedicinal uses, phytochemistry, pharmacological properties and toxicology. J. Ethnopharmacol. 2015, 165, 215–226. 10.1016/j.jep.2015.02.041. 25724970

[ref10] Watt J. M.; Breyer-Brandwijk M. G. The medicinal and poisonous plants of Southern and Eastern Africa: being an account of their medicinal and other uses. Chemical Composition, Pharmacological Effects and Toxicology in Man and Animal, 2nd ed.; Livingstone: Edinburgh, 1962.

[ref11] Muazu J.; Kaita M. A review of traditional plants used in the treatment of epilepsy amongst the Hausa/Fulani tribes of northern Nigeria. Afr. J. Tradit. Complement. Altern. Med. 2008, 5 (4), 387–390. 10.4314/ajtcam.v5i4.31294. 20161961 PMC2816574

[ref12] Kadiri A. B.; Agboola O. M.; Fashina F. O. Ethnobotanical survey and phyto-anatomical studies of some common plants used for the treatment of epilepsy in some rural areas of South west Nigeria. Pharmacogn. J. 2014, 6 (2), 17–23. 10.5530/pj.2014.2.3.

[ref13] Birhan Y. S. Medicinal plants utilized in the management of epilepsy in Ethiopia: ethnobotany, pharmacology and phytochemistry. Chin. Med. 2022, 17 (1), 129 10.1186/s13020-022-00686-5. 36403053 PMC9675240

[ref14] Okomolo F. C.; Mbafor J. T.; Bum E. N.; Kouemou N.; Kandeda A. K.; Talla E.; Dimo T.; Rakotonirira A.; Rakotonirira E. Evaluation of the sedative and anticonvulsant properties of three Cameroonian plants. Afr. J. Tradit. Complement. Altern. Med. 2011, 8 (5S), 181–190. 10.4314/ajtcam.v8i5s.24. 22754073 PMC3252709

[ref15] Maroyi A. Traditional use of medicinal plants in south-central Zimbabwe: review and perspectives. J. Ethnobiol. Ethnomed. 2013, 9 (1), 31 10.1186/1746-4269-9-31. 23642285 PMC3653698

[ref16] Kinda P. T.; Zerbo P.; Guenné S.; Compaoré M.; Ciobica A.; Kiendrebeogo M. Medicinal Plants Used for Neuropsychiatric Disorders Treatment in the Hauts Bassins Region of Burkina Faso. Medicines 2017, 4 (2), 32 10.3390/medicines4020032. 28930246 PMC5590068

[ref17] Adeyemi O. O.; Akindele A. J.; Yemitan O. K.; Aigbe F. R.; Fagbo F. I. Anticonvulsant, anxiolytic and sedative activities of the aqueous root extract of *Securidaca longepedunculata* Fresen. J. Ethnopharmacol. 2010, 130 (2), 191–195. 10.1016/j.jep.2010.04.028. 20435127

[ref18] Dibwe D. F.; Awale S.; Kadota S.; Morita H.; Tezuka Y. Heptaoxygenated xanthones as anti-austerity agents from *Securidaca longepedunculata*. Bioorg. Med. Chem. 2013, 21 (24), 7663–7668. 10.1016/j.bmc.2013.10.027. 24216090

[ref19] Joseph C. C.; Moshi M. J.; Sempombe J.; Nkunya M. H. H. (4-Methoxy-benzo[1,3]dioxol-5-yl)- *Phenylmethanone*: an antibacterial benzophenone from *Securidaca longepedunculata*. Altern. Med. 2006, 3 (3), 80–86. 10.4314/ajtcam.v3i3.31169.

[ref20] Wang Q.; Ma C.; Ma Y.; Li X.; Chen Y.; Chen J. Structure-activity relationships of diverse xanthones against multidrug resistant human tumor cells. Bioorg. Med. Chem. Lett. 2017, 27 (3), 447–449. 10.1016/j.bmcl.2016.12.045. 28065566

[ref21] Meyer J. M.; Rakuambo N. C.; Hussein A. A. Novel xanthones from *Securidaca longepedunculata* with activity against erectile dysfunction. J. Ethnopharmacol. 2008, 119 (3), 599–603. 10.1016/j.jep.2008.06.018. 18638534

[ref22] Rakuambo N. C.; Meyer J. J. M.; Hussein A. Xanthone isolated from *Securidaca longependunculata* with activity against erectile dysfunction. Fitoterapia 2004, 75 (5), 497–499. 10.1016/j.fitote.2004.03.010. 15261388

[ref23] Meli A. L.; Ngninzeko F. N.; Castilho P. C.; Wansi J. D.; Kuete V.; Lontsi D.; Beng V. P.; Choudhary M. I.; Sondengam B. L. Securidacaxanthones B and C, xanthones from *Securidaca longepedunculata* (Polygalaceae). Planta Med. 2007, 73 (09), 411 10.1055/s-2007-987191.

[ref24] Zuo J.; Xia Y.; Li X.; Chen J.-w. Xanthones from *Securidaca inappendiculata* exert significant therapeutic efficacy on adjuvant-induced arthritis in mice. Inflammation 2014, 37 (3), 908–916. 10.1007/s10753-014-9810-8. 24419745

[ref25] Banerjee P. S.; Sharma P. K. New antiepileptic agents: structure–activity relationships. Med. Chem. Res. 2012, 21 (7), 1491–1508. 10.1007/s00044-011-9615-3.

[ref26] Malawska B. New anticonvulsant agents. Curr. Top. Med. Chem. 2005, 5 (1), 69–85. 10.2174/1568026053386944. 15638779

[ref27] Sunderkötter C.; Wohlrab J.; Hamm H. Scabies: Epidemiology, Diagnosis, and Treatment. Dtsch. Arztebl. Int. 2021, 118 (41), 695–704. 34615594 10.3238/arztebl.m2021.0296 PMC8743988

[ref28] Neuwinger H. D. African Ethnobotany: Poisons and Drugs: Chemistry, Pharmacology, Toxicology; Chapman & Hall: London: Heidelberg, Germany, 1996.

[ref29] Jung J. H.; Pummangura S.; Chaichantipyuth C.; Patarapanich C.; McLaughlin J. L. Bioactive constituents of *Melodorum fruticosum*. Phytochemistry 1990, 29 (5), 1667–1670. 10.1016/0031-9422(90)80142-4.

[ref30] Ma W.-W.; Anderson J. E.; McLaughlin J. L. Bioactive Benzyl Benzoates from the Roots of *Endlicheria Dysodantha*. Int. J. Pharmacogn. 1991, 29 (3), 237–239. 10.3109/13880209109082886.

[ref31] Lu B.; Zhu F.; Sun H. M.; Shen Q. Esterification of the Primary Benzylic C-H Bonds with Carboxylic Acids Catalyzed by Ionic Iron(III) Complexes Containing an Imidazolinium Cation. Org. Lett. 2017, 19 (5), 1132–1135. 10.1021/acs.orglett.7b00148. 28198186

[ref32] Ekwy O. C.; Ehiabhi O. S.; Okechukwu E. B. Phytochemical and GC-MS analyses of the bioactive components of *Securidaca longepedunculata* (Fresen) roots for anti-breast cancer activity. World J. Pharm. Res. 2015, 4 (12), 1503–1518.

[ref33] Song M.-C.; Yang H.-J.; Baek N.-I. A New Benzophenone from *Lindera fruticosa*. Bull. Korean Chem. Soc. 2007, 28 (7), 1209–1210.

[ref34] Ito C.; Matsui T.; Niimi A.; Tan H. T.; Itoigawa M. Four New Xanthones from *Cratoxylum cochinchinense* and Their In Vitro Antiproliferative Effects. Planta Med. 2017, 83 (09), 812–818. 10.1055/s-0043-102510. 28158891

[ref35] Dao T. T.; Dang T. T.; Nguyen P. H.; Kim E.; Thuong P. T.; Oh W. K. Xanthones from *Polygala karensium* inhibit neuraminidases from influenza A viruses. Bioorg. Med. Chem. Lett. 2012, 22 (11), 3688–3692. 10.1016/j.bmcl.2012.04.028. 22552195

[ref36] Wedajo F.; Gure A.; Meshesha M.; Kedir K.; Frese M.; Sewald N.; Abdissa N. Cytotoxic compounds from the root bark of *Securidaca longipedunculata*. Bull. Chem. Soc. Ethiop. 2022, 36 (2), 417–422. 10.4314/bcse.v36i2.14.

[ref37] Galeffi C.; Federici E.; Msonthi J. D.; Marini-Bettolo G. B.; Nicoletti M. New xanthones from *Ectiadiopsis oblongifolia* and *Securidaca longipedunculata*. Fitoterapia 1990, 61 (1), 79–81.

[ref38] Meli Lannang A.; Lontsi D.; Ngounou F. N.; Sondengam B. L.; Nkengfack A. E.; van Heerden F. R.; Assob J. C. Securidacaxanthone A, a heptaoxygenated xanthone from *Securidaca longepedunculata*. Fitoterapia 2006, 77 (3), 199–202. 10.1016/j.fitote.2006.01.006. 16564647

[ref39] Iinuma M.; Tosa H.; Ito T.; Tanaka T.; Madulid D. A. Two xanthones from roots of *Cratoxylum formosanum*. Phytochemistry 1996, 42 (4), 1195–1198. 10.1016/0031-9422(96)00111-2.

[ref40] Zou J.; Jin D.; Chen W.; Wang J.; Liu Q.; Zhu X.; Zhao W. Selective cyclooxygenase-2 inhibitors from *Calophyllum membranaceum*. J. Nat. Prod. 2005, 68 (10), 1514–1518. 10.1021/np0502342. 16252917

[ref41] Zhang L.; Yang X.; Xu L.; Yang S. Three New Xanthones from the Roots of *Securidaca inappendiculata*. Heterocycles 2005, 65 (7), 1685–1690.

[ref42] Zhou L.; Yu X.; Geng Y.; Hua Y. Chemical constituents and bioactivities of xanthones from *Polygala sibirica* L. var. *megalopha*. Chem. Ind. For. Prod. 2017, 37 (2), 121–128.

[ref43] Kwon Y. S.; Park C.-B.; Lee S.-M.; Zee S.; Kim G.-E.; Kim Y.-J.; Sim H.-J.; Kim J.-H.; Seo J.-S. Proteomic analysis of zebrafish (*Danio rerio*) embryos exposed to benzyl benzoate. Environ. Sci. Pollut. Res. 2022, 30 (10), 26375–26386. 10.1007/s11356-022-24081-7. PMC9995408 36367642

[ref44] Burgess I. *Sarcoptes scabiei* and scabies. Adv. Parasitol. 1994, 33, 235–292. 10.1016/S0065-308X(08)60414-5. 8122567

[ref45] European Chemicals Agency. Benzyl benzoate. https://echa.europa.eu/substance-information/-/substanceinfo/100.004.003 (accessed August 08, 2023).

[ref46] Acar A.; Türkmen Z.; Çavuşoğlu K.; Yalçin E. Investigation of benzyl benzoate toxicity with anatomical, physiological, cytogenetic and biochemical parameters in in vivo. Caryologia 2020, 73, 21–32.

[ref47] Casu L.; Solinas M. N.; Saba A. R.; Cottiglia F.; Caboni P.; Floris C.; Laconi S.; Pompei R.; Leonti M. Benzophenones from the roots of the Popoluca Amerindian medicinal plant *Securidaca diversifolia* (L.) S.F. Blake. Phytochem. Lett. 2010, 3 (4), 226–229. 10.1016/j.phytol.2010.08.005.

[ref48] Zuo J.; Mao K.-j.; Yuan F.; Li X.; Chen J.-w. Xanthones with anti-tumor activity isolated from *Securidaca inappendiculata*. Med. Chem. Res. 2014, 23 (11), 4865–4871. 10.1007/s00044-014-1051-8.

[ref49] Lannang A. M.; Louh G. N.; Biloa B. M.; Komguem J.; Mbazoa C. D.; Sondengam B. L.; Naesens L.; Pannecouque C.; De Clercq E.; Sayed El Ashry E. H. Cytotoxicity of Natural Compounds Isolated from the Seeds of *Garcinia afzelii*. Planta Med. 2010, 76 (07), 708–712. 10.1055/s-0029-1240627. 19937549

[ref50] Ochora D. O.; Kakudidi E.; Namukobe J.; Heydenreich M.; Coghi P.; Yang L. J.; Mwakio E. W.; Andagalu B.; Roth A.; Akala H. M.; Wong V. K. W.; Yenesew A. A new benzophenone, and the antiplasmodial activities of the constituents of *Securidaca longipedunculata* fresen (Polygalaceae). Nat. Prod. Res. 2022, 36 (11), 2758–2766. 10.1080/14786419.2021.1925272. 34000936

[ref51] Dibwe D. F.; Awale S.; Kadota S.; Tezuka Y.; Muchimangins A.-D. novel diphenylmethyl-substituted xanthones from *Securidaca longepedunculata*. Tetrahedron Lett. 2012, 53 (46), 6186–6190. 10.1016/j.tetlet.2012.08.115.

[ref52] Noordin M. A. M.; Noor M. M.; Kamaruddin W. M. A. W.; Lazim A. M.; Fazry S. Toxicity test of xanthone from mangosteen on zebrafish embryos. AIP Conf. Proc. 2016, 1784 (1), 020014 10.1063/1.4966724.

[ref53] Fazry S.; Noordin M. A. M.; Sanusi S.; Noor M. M.; Aizat W. M.; Lazim A. M.; Dyari H. R. E.; Jamar N. H.; Remali J.; Othman B. A.; Law D.; Sidik N. M.; Cheah Y. H.; Lim Y. C. Cytotoxicity and Toxicity Evaluation of Xanthone Crude Extract on Hypoxic Human Hepatocellular Carcinoma and Zebrafish (*Danio rerio*) Embryos. Toxics 2018, 6 (4), 60 10.3390/toxics6040060. 30304811 PMC6316214

[ref54] Kittipaspallop W.; Taepavarapruk P.; Chanchao C.; Pimtong W. Acute toxicity and teratogenicity of α-mangostin in zebrafish embryos. Exp. Biol. Med. 2018, 243 (15–16), 1212–1219. 10.1177/1535370218819743. PMC6384447 30602309

[ref55] Kitipaspallop W.; Sillapaprayoon S.; Taepavarapruk P.; Chanchao C.; Pimtong W. Evaluation of developmental and transcriptional effects of α-mangostin on zebrafish embryos. Toxicol. Environ. Chem. 2021, 103 (3), 254–268. 10.1080/02772248.2021.1960349.

[ref56] Pimtong W.; Kitipaspallop W.; Chun H.-S.; Kim W.-K. Effects of α-mangostin on embryonic development and liver development in zebrafish. Mol. Cell Toxicol. 2020, 16 (4), 469–476. 10.1007/s13273-020-00099-1.

[ref57] Urbatzka R.; Freitas S.; Palmeira A.; Almeida T.; Moreira J.; Azevedo C.; Afonso C.; Correia-da-Silva M.; Sousa E.; Pinto M.; Vasconcelos V. Lipid reducing activity and toxicity profiles of a library of polyphenol derivatives. Eur. J. Med. Chem. 2018, 151, 272–284. 10.1016/j.ejmech.2018.03.036. 29626799

[ref58] Wang S.-n.; Li Q.; Jing M.-h.; Alba E.; Yang X.-h.; Sabaté R.; Han Y.-f.; Pi R.-b.; Lan W.-j.; Yang X.-b.; Chen J.-k. Natural Xanthones from *Garcinia mangostana* with Multifunctional Activities for the Therapy of Alzheimer’s Disease. Neurochem. Res. 2016, 41 (7), 1806–1817. 10.1007/s11064-016-1896-y. 27038926

[ref59] Sun C.; Li B.; Ma C.; Zhou Y. Application of 6-hydroxy-1,2,3,7-tetramethoxyxanthone in preparing drug for promoting hippocampal neurogenesis for treatment of depression. CN 10013476 A, 2019.

[ref60] Viewpoint-Behavior Technology. https://www.viewpoint.fr/product/zebrafish/fish-behavior-monitoring/zebralab (accessed June 19, 2023).

[ref61] Viewpoint Life Sciences Zebralab. High Throughput Monitoring of Fishes. https://m.ibric.org/miniboard/down.php?Board=new_protech&filename=Viewpoint%20Brochure.pdf&id=143531&fidx=1 (accessed December 20, 2023).

[ref62] Suls A.; Jaehn J. A.; Kecskés A.; Weber Y.; Weckhuysen S.; Craiu D. C.; Siekierska A.; Djémié T.; Afrikanova T.; Gormley P.; von Spiczak S.; Kluger G.; Iliescu C. M.; Talvik T.; Talvik I.; Meral C.; Caglayan H. S.; Giraldez B. G.; Serratosa J.; Lemke J. R.; Hoffman-Zacharska D.; Szczepanik E.; Barisic N.; Komarek V.; Hjalgrim H.; Møller R.; Linnankivi T.; Dimova P.; Striano P.; Zara F.; Marini C.; Guerrini R.; Depienne C.; Baulac S.; Kuhlenbäumer G.; Crawford A. D.; Lehesjoki A.-E.; de Witte P.; Palotie A.; Lerche H.; Esguerra C.; De Jonghe P.; Helbig I.; Hendrickx R.; Holmgren P.; Stephani U.; Muhle H.; Pendiziwiat M.; Appenzeller S.; Selmer K.; Brilstra E.; Koeleman B.; Rosenow F.; Leguern E.; Sterbova K.; Magdalena B.; Rodica G.; Arsene O.; Diana B.; Guerrero-Lopez R.; Ortega L.; Todorova A.; Kirov A.; Robbiano A.; Arslan M.; Yiş U.; Ivanović V.; Ivanović V. De Novo Loss-of-Function Mutations in CHD2 Cause a Fever-Sensitive Myoclonic Epileptic Encephalopathy Sharing Features with Dravet Syndrome. Am. J. Hum. Genet. 2013, 93 (5), 967–975. 10.1016/j.ajhg.2013.09.017. 24207121 PMC3824114

[ref63] McCarroll M. N.; Gendelev L.; Kinser R.; Taylor J.; Bruni G.; Myers-Turnbull D.; Helsell C.; Carbajal A.; Rinaldi C.; Kang H. J.; Gong J. H.; Sello J. K.; Tomita S.; Peterson R. T.; Keiser M. J.; Kokel D. Zebrafish behavioural profiling identifies GABA and serotonin receptor ligands related to sedation and paradoxical excitation. Nat. Commun. 2019, 10 (1), 4078 10.1038/s41467-019-11936-w. 31501447 PMC6733874

[ref64] Muto A.; Ohkura M.; Kotani T.; Higashijima S.; Nakai J.; Kawakami K. Genetic visualization with an improved GCaMP calcium indicator reveals spatiotemporal activation of the spinal motor neurons in zebrafish. Proc. Natl. Acad. Sci. U.S.A. 2011, 108 (13), 5425–5430. 10.1073/pnas.1000887108. 21383146 PMC3069178

[ref65] Randlett O.; Wee C. L.; Naumann E. A.; Nnaemeka O.; Schoppik D.; Fitzgerald J. E.; Portugues R.; Lacoste A. M.; Riegler C.; Engert F.; Schier A. F. Whole-brain activity mapping onto a zebrafish brain atlas. Nat. Methods 2015, 12 (11), 1039–1046. 10.1038/nmeth.3581. 26778924 PMC4710481

[ref66] Gawel K.; Langlois M.; Martins T.; van der Ent W.; Tiraboschi E.; Jacmin M.; Crawford A. D.; Esguerra C. V. Seizing the moment: Zebrafish epilepsy models. Neurosci. Biobehav. Rev. 2020, 116, 1–20. 10.1016/j.neubiorev.2020.06.010. 32544542

[ref67] Bandara S. B.; Carty D. R.; Singh V.; Harvey D. J.; Vasylieva N.; Pressly B.; Wulff H.; Lein P. J. Susceptibility of larval zebrafish to the seizurogenic activity of GABA type A receptor antagonists. Neurotoxicology 2020, 76, 220–234. 10.1016/j.neuro.2019.12.001. 31811871 PMC6957304

[ref68] Challal S.; Skiba A.; Langlois M.; Esguerra C. V.; Wolfender J.-L.; Crawford A. D.; Skalicka-Woźniak K. Natural product-derived therapies for treating drug-resistant epilepsies: From ethnopharmacology to evidence-based medicine. J. Ethnopharmacol. 2023, 317, 116740 10.1016/j.jep.2023.116740. 37315641

[ref69] Wong K.; Stewart A.; Gilder T.; Wu N.; Frank K.; Gaikwad S.; Suciu C.; Dileo J.; Utterback E.; Chang K.; Grossman L.; Cachat J.; Kalueff A. V. Modeling seizure-related behavioral and endocrine phenotypes in adult zebrafish. Brain Res. 2010, 1348, 209–215. 10.1016/j.brainres.2010.06.012. 20547142

[ref70] Baraban S. C.; Taylor M. R.; Castro P. A.; Baier H. Pentylenetetrazole induced changes in zebrafish behavior, neural activity and c-fos expression. Neuroscience 2005, 131 (3), 759–768. 10.1016/j.neuroscience.2004.11.031. 15730879

[ref71] Hostettmann K.; Hostettmann M.; Marston A. Preparative Chromatography Techniques: Applications in Natural Product Isolation; Springer-Verlag: Berlin, 1986.

[ref72] Nakonieczna S.; Grabarska A.; Gawel K.; Wróblewska-Łuczka P.; Czerwonka A.; Stepulak A.; Kukula-Koch W. Isoquinoline Alkaloids from Coptis chinensis Franch: Focus on Coptisine as a Potential Therapeutic Candidate against Gastric Cancer Cells. Int. J. Mol. Sci. 2022, 23 (18), 10330 10.3390/ijms231810330. 36142236 PMC9499618

[ref73] Aleström P.; D’Angelo L.; Midtlyng P. J.; Schorderet D. F.; Schulte-Merker S.; Sohm F.; Warner S. Zebrafish: Housing and husbandry recommendations. Lab. Anim. 2020, 54 (3), 213–224. 10.1177/0023677219869037. 31510859 PMC7301644

[ref74] Tiraboschi E.; Martina S.; van der Ent W.; Grzyb K.; Gawel K.; Cordero-Maldonado M. L.; Poovathingal S. K.; Heintz S.; Satheesh S. V.; Brattespe J.; Xu J.; Suster M.; Skupin A.; Esguerra C. V. New insights into the early mechanisms of epileptogenesis in a zebrafish model of Dravet syndrome. Epilepsia 2020, 61 (3), 549–560. 10.1111/epi.16456. 32096222

[ref75] Fleming A.; Diekmann H.; Goldsmith P. Functional characterisation of the maturation of the blood-brain barrier in larval zebrafish. PLoS One 2013, 8 (10), e77548 10.1371/journal.pone.0077548. 24147021 PMC3797749

[ref76] Quiñonez-Silvero C.; Hübner K.; Herzog W. Development of the brain vasculature and the blood-brain barrier in zebrafish. Dev. Biol. 2020, 457 (2), 181–190. 10.1016/j.ydbio.2019.03.005. 30862465

[ref77] Guo S. Using zebrafish to assess the impact of drugs on neural development and function. Expert Opin. Drug Discovery 2009, 4 (7), 715–726. 10.1517/17460440902988464. PMC2747263 19774094

[ref78] Westerfield M. The Zebrafish Book A Guide for the Laboratory Use of Zebrafish (Danio rerio). 5th ed.; University of Oregon Press: Eugene, 2007.

[ref79] Afrikanova T.; Serruys A. S.; Buenafe O. E.; Clinckers R.; Smolders I.; de Witte P. A.; Crawford A. D.; Esguerra C. V. Validation of the zebrafish pentylenetetrazol seizure model: locomotor versus electrographic responses to antiepileptic drugs. PLoS One 2013, 8 (1), e54166 10.1371/journal.pone.0054166. 23342097 PMC3544809

[ref80] He J.-H.; Guo S.-Y.; Zhu F.; Zhu J.-J.; Chen Y.-X.; Huang C.-J.; Gao J.-M.; Dong Q.-X.; Xuan Y.-X.; Li C.-Q. A zebrafish phenotypic assay for assessing drug-induced hepatotoxicity. J. Pharmacol. Toxicol. Methods 2013, 67 (1), 25–32. 10.1016/j.vascn.2012.10.003. 23128142

[ref81] Gawel K.; Turski W. A.; van der Ent W.; Mathai B. J.; Kirstein-Smardzewska K. J.; Simonsen A.; Esguerra C. V. Phenotypic characterization of larval zebrafish (*Danio rerio*) with partial knockdown of the cacna1a gene. Mol. Neurobiol. 2020a, 57 (4), 1904–1916. 10.1007/s12035-019-01860-x. 31875924 PMC7118054

[ref82] Berghmans S.; Hunt J.; Roach A.; Goldsmith P. Zebrafish offer the potential for a primary screen to identify a wide variety of potential anticonvulsants. Epilepsy Res. 2007, 75 (1), 18–28. 10.1016/j.eplepsyres.2007.03.015. 17485198

[ref83] Orellana-Paucar A. M.; Serruys A.-S. K.; Afrikanova T.; Maes J.; De Borggraeve W.; Alen J.; León-Tamariz F.; Wilches-Arizábala I. M.; Crawford A. D.; de Witte P. A. M.; Esguerra C. V. Anticonvulsant activity of bisabolene sesquiterpenoids of *Curcuma longa* in zebrafish and mouse seizure models. Epilepsy Behav. 2012, 24 (1), 14–22. 10.1016/j.yebeh.2012.02.020. 22483646

