# Supplementary material for: Inhibition of Seizure-Like Paroxysms and Toxicity Effects of Securidaca longepedunculata Extracts and Constituents in Zebrafish Danio rerio
Source: ACS Chem Neurosci. 2024 Jan 25;15(3):617–28. doi: 10.1021/acschemneuro.3c00642 (PMC10853935; doi:10.1021/acschemneuro.3c00642)
Supplement: Supplementary file 1 — cn3c00642_si_001.pdf [file cn3c00642_si_001.pdf]

Supporting information

Inhibition of seizure-like paroxysms and toxicity effects of *Securidaca longepedunculata* extracts and constituents in zebrafish *Danio rerio*

Nastaran Moussavi[1], Wietske van der Ent[2], Drissa Diallo[3,4†], Rokia Sanogo[3,4], Karl E. Malterud[1], Camila V. Esguerra[5**], Helle Wangensteen[1*, **]

[1] Section for Pharmaceutical Chemistry, Department of Pharmacy, University of Oslo, P.O. Box 1068, 0316 Oslo, Norway

[2] NCMM, Chemical Neuroscience Group, Centre for Molecular Medicine Norway, Faculty of Medicine, University of Oslo, 0349 Oslo, Norway

[3] Department of Traditional Medicine, National Institute of Public Health, PB 1746, Bamako, Mali

[4] Faculty of Pharmacy, University of Sciences, Techniques and Technologies of Bamako (USTTB), Bamako, Mali

[5] Section for Pharmacology and Pharmaceutical Biosciences, Department of Pharmacy, University of Oslo, P.O. Box 1068, 0316 Oslo, Norway

*Corresponding author:

Helle Wangensteen

P.O. Box 1068 Blindern

0316 Oslo

Norway

E-mail: helle.wangensteen@farmasi.uio.no

**Shared authorship

† Deceased

Locomotor activity tracking assay *(in vivo)*

- **Habituation:** wait 15 min

↓

- **PTZ (pentylenetetrazol) – a chemoconvulsant:**
  - **- stock:** 60 mM
  - **- final concentration:** 20 mM

↓

- **PTZ activation time:** wait 5 min

↓

- **Recording of larval movement** (measured as distance travelled)
  - **- Black:** inactive **< 4 mm/sec**
  - **- Green:** small **4-20 mm/sec**
  - **- Red:** Large **> 20 mm/sec**

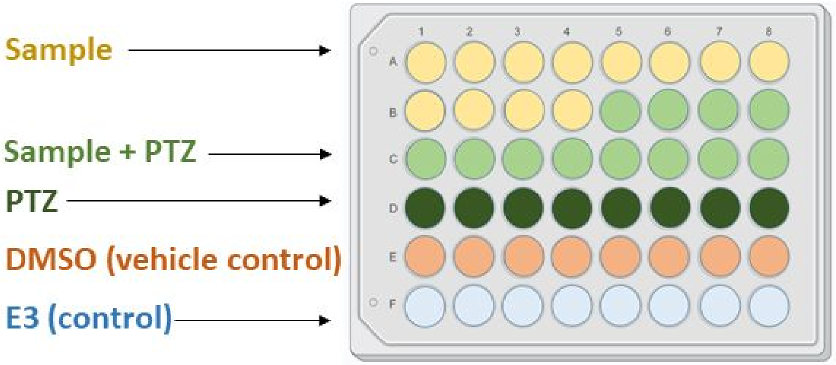

After PTZ-addition:

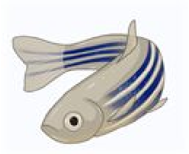

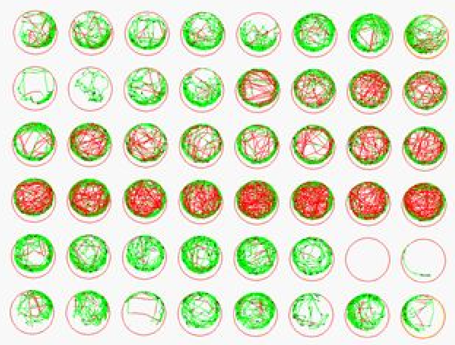

DCM extract **(0-300 s = 5 min, first time bin):**

NMR data for isolated compounds

1, benzyl benzoate (CAS 120-51-4)

^{1}H NMR ($CDCl_3$): 8.08 (2H, d, J 8.5 Hz), 7.51-7.57 (1H, t, J 7.4 Hz), 7.32-7.48 (7H, m), 5.37 (2H, s).
^{13}C NMR ($CDCl_3$): 166.5, 136.1, 133.1, 130.2, 129.7, 128.6, 128.4, 128.3, 66.7.
(Literature data: Lu et al 2017).

2, benzyl 2-hydroxy-6-methoxybenzoate (CAS 24474-71-3)

^{1}H NMR ($CDCl_3$): 11.41 (1H, s, OH), 7.45-7.49 (2H, m), 7.30-7.41 (4H, m), 6.59 (1H, dd, J 1.0 and 8.4 Hz), 6.41 (H, dd, J 1.0 and 8.4 Hz), 5.42 (2H, s), 3.85 (3H, s, OCH_3).
^{13}C NMR ($CDCl_3$): 170.9, 163.7, 161.1, 135.8, 135.3, 128.5, 128.1, 127.5, 110.1, 102.4, 66.9, 56.2.
(Literature data: Choi et al 2005).

3, 4-hydroxy-2,3-dimethoxybenzophenone (CAS 872881-75-9)

^{1}H NMR ($CDCl_3$): 7.78-7.87 (2H, m), 7.54-7.57 (1H, m), 7.45-7.47 (2H, m), 7.08 (1H, d, J 8.5 Hz), 6.77 (1H, d, J 8.5 Hz), 3.96 (3H, s, OCH~~3~~), 3.74 (3H, s, OCH_3).
^{13}C NMR ($CDCl_3$): 195.3, 153.1, 152.0, 139.6, 138.4, 132.8, 129.8, 128.2, 126.0, 125.6, 110.1, 61.5, 61.1.
(Literature data: Chong et al 2007)

4, 4,8-dihydroxy-1,2,3,5,6-pentamethoxyxanthone (CAS 1516880-35-5)

^{1}H NMR ($CDCl_3$): 13.17 (1H, br s, OH); 6.38 (1H, s), 4.12 (3H, s, OCH_3); 4.01 (3H, s, OCH_3); 3.98 (3H, s, OCH_3); 3.95 (3H, s, OCH_3); 3.94 (3H, s, OCH_3).
^{13}C NMR ($CDCl_3$): 180.5, 159.3, 159.2, 148.7, 148.6, 137.8, 131.5, 128.3, 103.1, 94.9, 62.1, 61.8, 61.7, 61.5, 56.3.
(Literature data: Dibwe et al 2013).

5, 1,6-dihydroxy-2,7,8-trimethoxyxanthone (CAS 2226450-52-6)

^{1}H NMR ($CDCl_3$): 13.33 (1H, s, OH), 7.23 (1H, d, J 9.0 Hz), 6.80 (1H, d, J 9.0 Hz), 6.74 (1H, s), 4.02 (3H, s, OCH_3), 4.02 (3H, s, OCH_3), 3.93 (3H, s, OCH_3).
^{13}C NMR ($CDCl_3$): 181.9, 155.9, 154.9, 152.2, 150.9, 149.1, 142.7, 137.2, 119.7, 108.9, 108.7, 104.7, 98.8, 61.9, 61.7, 57.1.
(Literature data: Ito et al 2017).

6, 1,6,8-trihydroxy-2,3,4,5-tetramethoxyxanthone (CAS 1516880-31-1)

^{1}H NMR ($CDCl_3$): 11.86 (1H, s), 11.81 (1H, s), 6.41 (1H,s), 4.15 (3H, s, OCH_3), 4.09 (3H, s, OCH_3), 3.97 (3H, s, OCH_3), 3.94 (3H, s, OCH_3).
^{13}C NMR ($CDCl_3$): 184.0, 158.4, 156.7, 154.5, 150.6, 148.1, 145.2, 136.1, 132.7, 126.8, 103.3, 101.7, 98.1, 62.0, 61.8, 61.7, 61.3.
(Literature data: Dibwe et al 2013).

7, 1,7-dihydroxy-4-methoxyxanthone (CAS 87339-76-2)

^{1}H NMR ($CDCl_3$): 12.04 (1H, s), 7.63 (1H, d, J 3.0 Hz), 7.54 (1H, d, J 9.0 Hz), 7.33 (1H, dd, J 9.0, 3.1 Hz), 7.26 (1H, d, J 8.9 Hz), 6.74 (1H, d, J 8.9 Hz), 3.97 (3H, s).
^{13}C NMR (acetone-d6): 182.9, 155.3, 155.1, 151.0, 146.9, 141.2, 126.2, 121.9, 121.2, 120.4, 109.7, 108.8, 57.3,
(Literature data: Dao et al 2012, Marston et al 1993).

8, 2-hydroxy-1,7-dimethoxyxanthone (CAS 872881-76-0)

^{1}H NMR ($CDCl_3$): 7.68 (1H, d, J 3.1 Hz), 7.40 (1H, d, J 9.2 Hz), 7.38 (1H, d, J 9.1 Hz), 7.31 (1H, dd, J 9.1, 3.1 Hz), 7.23 (1H, d, J9.1 Hz), 5.97 (1H, br s), 4.05 (3H, s), 3.92 (3H, s).
^{13}C NMR ($CDCl_3$): 176.1, 157.9, 150.3, 145.0, 124.8, 122.2, 119.0, 114.2, 105.6, 62.6, 55.9,
(Literature data: Galeffi et al 1990).

9, 2,7-dihydroxy-1,8-dimethoxyxanthone (CAS 178405-51-1)

^{1}H NMR ($CDCl_3$): 7.36 (2H, d, J 9.1 Hz), 7.16 (2H, d, J 9.1 Hz), 3.97 (6H, s).
^{13}C NMR ($CDCl_3$): 150.4, 145.1, 144.2, 122.2, 113.8, 62.6.
(Literature data: Iinuma et al 1996).

10, 3,7-dihydroxy-1,2,8-trimethoxyxanthone (CAS 863308-71-8)

^{1}H NMR ($CDCl_3$): 7.31 (1H, d, J 9.0 Hz), 7.12 (1H, d, J 9.0 Hz), 6.73 (1H, s), 6.46 (1H, br s, OH), 5.97 (1H, br s, OH), 4.04 (3H, s, OCH_3), 4.03 (3H, s, OCH_3), 4.01 (3H, s, OCH_3).
^{13}C NMR ($CDCl_3$): 175.1, 154.6, 154.0, 150.1, 145.3, 144.4, 137.1, 129.0, 122.7, 118.1, 113.4, 98.9, 62.7, 61.9, 61.8.
(Literature data: Zhang et al 2005).

References

Choi, S.Z., Choi, S.U., Bae, S.Y., Pyo, S.N. & Lee, K.R. (2005). Immunobiological Activity of a New Benzyl Benzoate from the Aerial Parts of *Solidago virga-aurea* var. *gigantea*. *Archives of Pharmacal Research 28*(1), 49-54. https://link.springer.com/article/10.1007/BF02975135

Chong, M.-C., Nigussie, F., Yang, H.-J. & Bek, N.-I. (2007) A new benzophenone from *Lindera fruticosa*. *Bulletin of the Korean Chemical Society 28* , 1209-1210. http://koreascience.or.kr/article/JAKO200702727454943.pdf

Dao, T.T., Dang, T.T., Nguyen, P.H., Kim, E., Thuong, P.T. & Oh, W.K. (2012) Xanthones from *Polygala karensium* inhibit neuraminidases from influenza A viruses. *Bioorganic & Medicinal Chemistry Letters 22*(11), 3688-3692. https://www.sciencedirect.com/science/article/pii/S0960894X12004672

Dibwe, D.F., Awale, S., Kadota, S., Morita, H. & Tezuka, Y. (2013) Heptaoxygenated xanthones as anti-austerity agents from *Securidaca longepedunculata*. *Bioorganic & Medicinal Chemistry 21*(24), 7663-7668. https://www.sciencedirect.com/science/article/pii/S0968089613008924?via%3Dihub

Galeffi, C., Federic,i E., Msonthi, J.D., Marini-Bettolo, G.B. & Nicoletti,. M (1990) New xanthones from *Ectiadiopsis oblongifolia* and *Securidaca longipedunculata*. *Fitoterapia 61*(1), 79-81.

Iinuma, M., Tosa, H., Ito, T., Tanaka, T. & Madulid, D.A. (1996) Two xanthones from roots *of Cratoxylum formosanum*. *Phytochemistry 42*(4) , 1195-1198. https://www.sciencedirect.com/science/article/pii/0031942296001112

Ito, C., Matsu,i T., Niimi, A. & Itoigawa, M. (2017) Four new xanthones from *Cratoxylum cochinchinense. Planta Medica 83*(9), 812-818. https://www.thieme-connect.de/products/ejournals/abstract/10.1055/s-0043-102510

Lu, B., Zhu, F., Sun, H.-M. & Shen, Q. (2017) Esterification of the Primary Benzylic C−H Bonds with Carboxylic Acids Catalyzed by Ionic Iron(III) Complexes Containing an Imidazolinium Cation. *Organic Letters 19*(5), 1132-1135.
https://pubs.acs.org/doi/10.1021/acs.orglett.7b00148

Marston, A., Hamburger, M., Sordat-Diserens, I., Msonthi, J.D. & Hostettmann, K. (1993) Xanthones from *Polygala nyikensis*. *Phytochemistry 33*(4), 809-812. https://www.sciencedirect.com/science/article/pii/003194229385279Z

Zhang L-j, Yang X-d, Xu L-z, Yang S-l (2005) Three new xanthones from the roots of *Securidaca inappendiculata*. *Heterocycles 65*(7), 1685-1690.
https://cir.nii.ac.jp/crid/1523669555536648448
